# Finite Element Analysis of Glass Fiber-Reinforced Polymer-(GFRP) Reinforced Continuous Concrete Beams

**DOI:** 10.3390/polym13244468

**Published:** 2021-12-20

**Authors:** Hazem Ahmad, Amr Elnemr, Nazam Ali, Qudeer Hussain, Krisada Chaiyasarn, Panuwat Joyklad

**Affiliations:** 1 Civil Engineering Department, German University in Cairo (GUC), New Cairo City 11835, Egypt; hazem.3008@gmail.com; 2 Material Engineering at Civil Engineering Department, German University in Cairo (GUC), New Cairo City 11835, Egypt; amr.elnemr@guc.edu.eg; 3 Department of Civil Engineering, School of Engineering, University of Management and Technology, Lahore 54770, Pakistan; nazam.ali@umt.edu.pk; 4 Center of Excellence in Earthquake Engineering and Vibration, Department of Civil Engineering, Chulalongkorn University, Bangkok 10330, Thailand; ebbadat@hotmail.com; 5 Thammasat Research Unit in Infrastructure Inspection and Monitoring, Repair and Strengthening (IIMRS), Thammasat School of Engineering, Faculty of Engineering, Thammasat University Rangsit, Pathumthani 12121, Thailand; ckrisada@engr.tu.ac.th; 6 Department of Civil and Environmental Engineering, Faculty of Engineering, Srinakharinwirot University, Nakhon Nayok 26120, Thailand

**Keywords:** concrete, glass fiber-reinforced polymer, stirrups, continuous-beam, moment redistribution, shear capacity

## Abstract

Fiber-reinforced concrete (FRC) is a competitive solution for the durability of reinforced structures. This paper aims to observe the moment redistribution behavior occurring due to flexural and shear loading in Glass Fiber-Reinforced Polymer- (GFRP) reinforced continuous concrete beams. A rectangular cross-section was adopted in this study with dimensions of 200 mm in width and 300 mm in depth with a constant shear span-to-depth ratio of 3. The reinforcement ratio for the top and bottom were equal at sagging and hogging moment regions. A finite element model was created using Analysis System (ANSYS) and validated with the existing experimental results in the literature review. Based on the literature review, the parametric study was conducted on twelve beam specimens to evaluate the influence of concrete compressive strength, transversal GFRP stirrups ratio, and longitudinal reinforcement ratio on the redistribution of the moment in beams. Several codes and guidelines adopted different analytical models. The Canadian Standards Association (CSA) S806 adopted the modified compression field theory in predicting the shear capacity of the simply supported beams. Recently, various researchers encountered several factors and modifications to account for concrete contribution, longitudinal, and transverse reinforcement. A comparison between the predicting shear capacity of the generated finite element model, the analytical model, and the existing data from the literature was performed. The generated finite element model showed a good agreement with the experimental results, while the beam specimens failed in shear after undergoing significant moment redistribution from hogging to sagging moment region. The moment distribution observed about 21.5% from FEM of beam specimen GN-1.2-0.48-d, while the experimental results achieved 24% at failure load. For high strength concrete presented in beam specimen GH-1.2-0.63-d, the result showed about 20.2% moment distribution, compared to that achieved experimentally of 23% at failure load.

## 1. Introduction

In aggressive marine environments or structures persistently exposed to corrosive elements, the deterioration of RC (reinforced concrete) structures is a significant problem, particularly the corrosion of steel reinforcement bars. Recently FRP (Fiber-Reinforced Polymer) composites have been used for strengthening of concrete structures [1,2]. Also, FRP has been introduced to replace steel reinforcement to overcome this problem. The critical drawback in the FRP bars presented is limiting the plastic response of the RC structures, due to the brittleness provided by their linear elastic behavior until failure. Moreover, GFRP (Glass Fiber-Reinforced Polymer) has a considerably lower elastic modulus than steel [3]. Moment redistribution occurs in continuous beams, typically due to extensive cracking and in the plastic hinge formation at middle support after top steel reinforcement yields. Consequently, the beam behaves less continuously and more as simply supported and redistributes the acting forces and moments away from the middle support to mid-span regions. The difference between the bending moments from elastic beam analysis and the actual moment could be defined as the moment redistribution. The FRP, including but not limited to the GFRP, lacks the plastic behavior of steel reinforcement at failure and instead fails in a brittle manner. Thus, the moment redistribution behavior for RC structures using longitudinal and transverse FRP bars required investigation [4,5]. 

Several studies confirmed that FRP reinforced continuous beams redistributed moments similar to that in steel-reinforced continuous beams [6,7,8]. Mahmoud and El-Salakawy [9] and El-Mogy et al. [10,11] reported that FRP–RC beams redistributed moments due to FRP bars’ bond-slip behavior, large strains at failure, and the inelasticity of concrete. Their experimental results concluded that FRP-reinforced continuous beams redistributed bending moments by at least 20%. Furthermore, the results showed that increasing the transverse reinforcement spacing and constant transverse reinforcement ratio led to more moment redistribution. The influence of transverse reinforcement on moment redistribution could explain the lower stiffness resulting from the lower GFRP’s elastic modulus. In RC beams of critical shear forces, increasing the GFRP transverse reinforcement ratio causes an increase in diagonal cracking but with lower crack widths [12]. Shehata [13] and Ahmed et al. [14] urged that the lower tensile strength be assigned for the bent portions of the FRP stirrup than for the straight portion. Their hypothesis denoted that the anticipated shear failure usually occurs in the bent portion of the FRP stirrups. Thus, the American Concrete Institute (ACI) 440.1R [15] and Canadian Standards Association (CSA) S806 [16] limited the FRP stirrups’ strength by 4000 microstrains to account for the lower tensile strength of the bent portion for the FRP stirrup and limited the shear cracking. 

Studies investigating the moment redistribution of beams typically use beams that are flexure-critical [17,18,19]. Thus, they fail in flexural compression or tension before failing in shear. Other numerical techniques involving deducing moment redistribution through member stiffness deduced from cross-section moment-curvature relationship neglects or does not adequately consider the shear stiffness of beam and, hence, its impact on the moment redistribution of the beam [6,20]. With these factors in place, the formation of diagonal shear crack at both interior and exterior support should cause considerable stiffness losses at its region, as the overall stiffness is resisted by GFRP stirrups and involved in the shear transfer mechanism at the site of the crack. Alam et al. [21] studied the modeling behavior of GFRP longitudinally reinforced single span concrete members using the ABAQUS package and compared the findings with GFRP reinforced beams. The modeling revealed that GFRP reinforced concrete beams showed similar shear behavior with a reasonable degree of accuracy. This behavior requires investigation into the impact of using the GFRP stirrups on the shear behavior of beams, their moment of redistribution, and the reliability of finite element modelling. In the past, the use of GFRP reinforced beams, slabs, and columns have been extensively studied both through laboratory investigations and finite element analysis [22,23]. Moreover, the bending behavior of precast concrete slabs with externally flanged hollow FRP tubes has been studied [24]. However, no studies have been published on the GFRP reinforced continuous concrete beams, in particular, where there is concern about the moment distribution under different types of loadings such as flexure and shear. Therefore, this paper aims to observe moment redistribution behavior occurring due to flexural and shear loading in Glass Fiber Reinforced Polymer GFRP reinforced continuous concrete beams. A rectangular cross-section was adopted in this study with dimensions of 200 mm in width and 300 mm in depth with a constant shear span-to-depth ratio of 3. The reinforcement ratio for the top and bottom were equal at sagging and hogging moment regions. A finite element model was created using Analysis System (ANSYS) and validated with the existing experimental results in the literature review. Based on the literature review, the parametric study was conducted on twelve beam specimens to evaluate the influence of concrete compressive strength, transversal GFRP stirrups ratio, and longitudinal reinforcement ratio on the redistribution of the moment in beams.

## 2. Methodology

**Figure 1:**
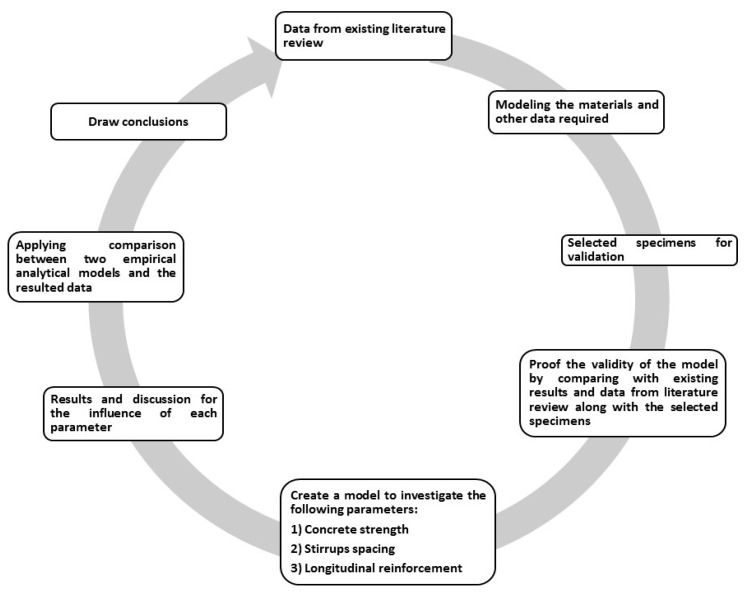
Typical schematic flowchart shows the sequence of the methodology adopted here in this study.

The methodology adopted here in this study is illustrated in the flowchart shown in Figure 1. From Figure 1, the methodology followed the sequence of searching the existing literature for the experimental work performed on continuous beams reinforced with FRP bars, including the fiber type, concrete grade, and shear capacities expected. Then, the study began to discuss the modelling of materials. From the literature specified, selected specimens were considered; materials and experimental testing were discussed under the section of numerical modelling using ANSYS [20]. While discussing the numerical modelling, convergence and meshing were also studied to check the results’ appropriateness. Then, the model was validated compared to two chosen experimental specimens, and the results were compared in terms of ultimate shear capacity and load-deflection curves. After the check on the model’s validity, a set of parameters were considered from the existing literature that could significantly influence the shear capacity and moment redistribution of the beams. The parameters were various concrete strengths varied incrementally by 10 MPa ranging from 30 to 80 MPa, stirrups spacing, and longitudinal reinforcement. After discussing the results of these parameters, two analytical models were introduced to validate the possibility of estimating the shear capacity of continuous beams reinforced with FRP bars, and a conclusion was drawn. The selected parameters were based on the current literature findings, as most results revealed that these are the main influencing parameters. The authors would like to highlight that even the modified compression theory would confirm the selected parameters. The CSA/S806 [16] provided that the concrete strength, stirrups spacing, and longitudinal would be the most influencing parameters. 

## 3. Selection of Constitutive Models

### 3.1. Concrete

An eight-node solid element, SOLID65, available in the ANSYS [20] element library, was adopted to model the concrete. The solid element has three translation degrees of freedom at each nodal direction; x, y, and z. The element could plastically deform, crack in three orthogonal directions, crush in compression, and exhibit creep behavior. The element could model the concrete either with rebar or without rebars included. For normal and high strength concrete, the modulus of elasticity, *E_c_* for concrete was calculated using Equations (1) and (2), respectively, while the uni-axial rupture stress, *f_r_*, was calculated using Equation (3).

(1) $$E_{c}=4500\sqrt{f^{'}}$$

(2) $$E_{c}=(3300\sqrt{f^{'}}+6900){\left(\frac{\gamma_{c}}{2300}\right)}^{1.5}$$

(3) $$f_{r}=0.6\sqrt{f^{'}}$$

where *f*′ is the concrete ultimate compressive strength, and *γ_c_* is the concrete density. The stress-strain relationships were calculated by Equations (4)–(6) [23,24,25].

(4) $$f=\frac{n\frac{\varepsilon }{\varepsilon_{0}}f}{n-1+{\left(\frac{\varepsilon }{\varepsilon_{0}}\right)}^{n}}$$

(5) $$\varepsilon_{0}=\frac{f^{\prime }}{E_{c}}\;\left(\frac{n}{n-1}\right)$$

(6) $$n=0.8+\left(\frac{f^{\prime }}{2500}\right)$$

where ε is the concrete strain,$\;\varepsilon_{0}$ is the concrete strain at its compressive strength; *f*′, and *n* is a curve-fitting factor. Furthermore, the open and closed shear transfer coefficients were assigned as 0.2 and 0.8. Finally, the Poisson’s ratio of concrete was assigned by 0.2 based on the current literature review [11,26,27,28], which agreed that the concrete’s Poisson ratio should donate by 0.2 when defining concrete inside the finite element software such as ANSYS. 

### 3.2. GFRP Reinforcement

A LINK180 element was used to model GFRP reinforcement as discrete elements with shared nodes to simulate the embedded reinforcement within the concrete section. The element has two nodes with three translational degrees of rotation, which presents the GFRP reinforcement embedded into the concrete section. This element is defined by linear-elastic behavior up to failure. Similar to the concrete, the Poisson’s ratio was valued by 0.2 for the GFRP reinforcement. Table 1 presents the properties and the details of the bar diameter adopted here in this study. It should be mentioned that the GFRP stirrups were included as one of the parameters set in this study. Moreover, the stirrups’ tensile strength was limited by 5000 micro strains as per CSA/S806 [16], which stated that the strength reduction should be maintained due to the stirrups’ bent portions.

### 3.3. The Bond between Concrete and Reinforcing Bars

**Figure 2:**
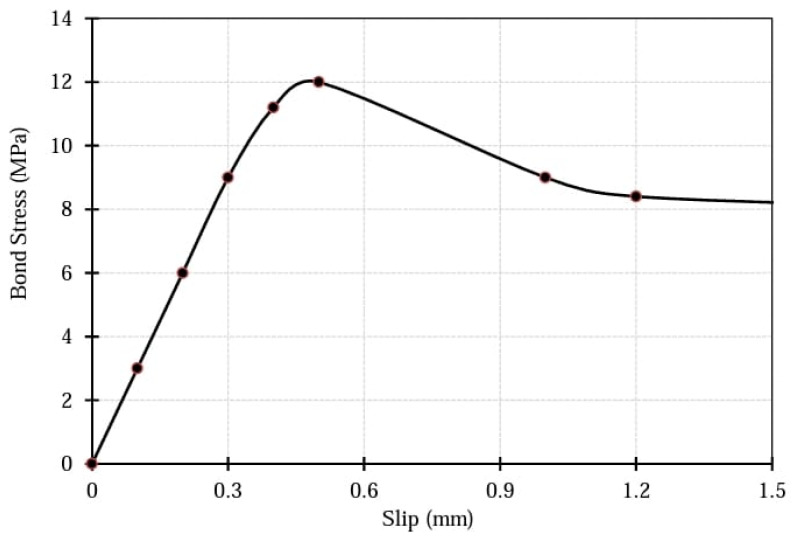
Bond Stress-Slip Model for sand-coated GFRP bars [29].

The elements of the GFRP reinforcement were at the exact location as those of the concrete elements. In other words, the LINK180 element, which shows the reinforcement, shares some nodes of SOLID65 that presents the concrete. A three uni-directional nonlinear spring element COMBIN39 was then used to simulate the embedding of the reinforcement within concrete elements in x, y, and z directions. The spring elements in the longitudinal direction, parallel to rebar reinforcement, denoted the reinforcement and concrete bond-slip behavior. The spring elements in the other two transverse directions, perpendicular to longitudinal reinforcement, exemplified the reinforcement’s dowel strength or anchorage reinforcement behavior. The spring force and the corresponding bond slip in the direction parallel to reinforcement were obtained from the bond stress and slip for sand-coated GFRP bars existing in the literature review. Alves et al. [29] investigated the influence of several parameters on the bond-slip behavior of the GFRP reinforcement, as shown in Figure 2. Figure 2 shows the bond stress-slip behavior adopted here in this study, simulating the GFRP reinforcement and concrete bond behavior.

Moreover, the anchorage behavior was modelled through the spring perpendicular to the direction of the reinforcement elements using the linear stress–displacement relationship. The stress was limited to the shear strength capacity of the reinforcement, while the corresponding displacement was evaluated by calculating the transverse strain of the reinforcement. The reinforcement’s transverse strain could be obtained by multiplying the longitudinal strain of the reinforcement with Poisson’s ratio. Thus, the corresponding displacement could be determined through transverse strain multiplied by the bar diameter. Although the discussion above regards the used properties for simulating the longitudinal and transverse GFRP reinforcement, the shear strength or dowel strength of bars was not mentioned in the experimental setup [26]. Thus, for the shear or dowel strength of GFRP reinforcement, a conservative assumption of 100 MPa for the shear strength per GFRP bar was assumed throughout the numerical modelling.

## 4. Model Calibration

**Figure 3:**
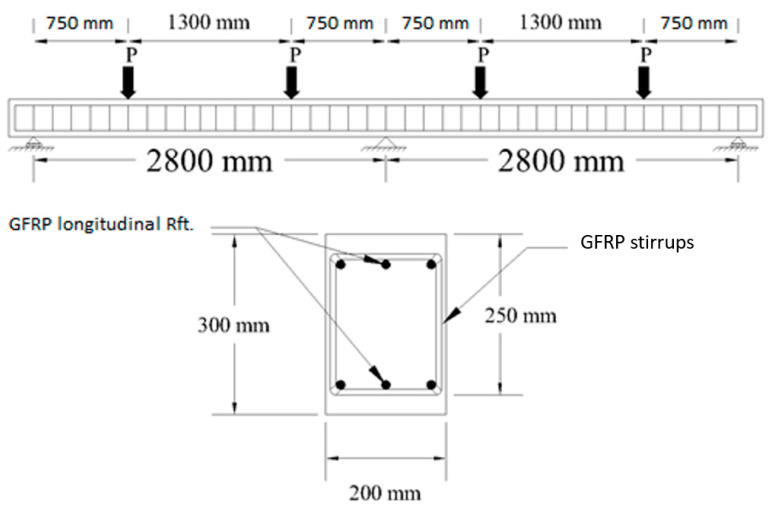
Dimensions, reinforcement details, and test setup of the beams used for verification by FEM.

Twelve continuous concrete beams reinforced by GFRP bars both longitudinally and transversely were analyzed using nonlinear finite element modeling using ANSYS [20]. The beam specimens had a cross-section of 200 mm in width and 300 mm in-depth, with every span having a length of 2800 mm. The beam specimens were exposed to two concentrated forces on each span of the specimen. The loading condition adopted was in conjunction with the design requirements assigned by CSA S806 [16] to undergo shear failure with a constant shear span-to-depth ratio of 3. Figure 3 shows the typical cross-section of beam specimens and the experimental setup adopted from the existing literature review [26].

**Figure 4:**
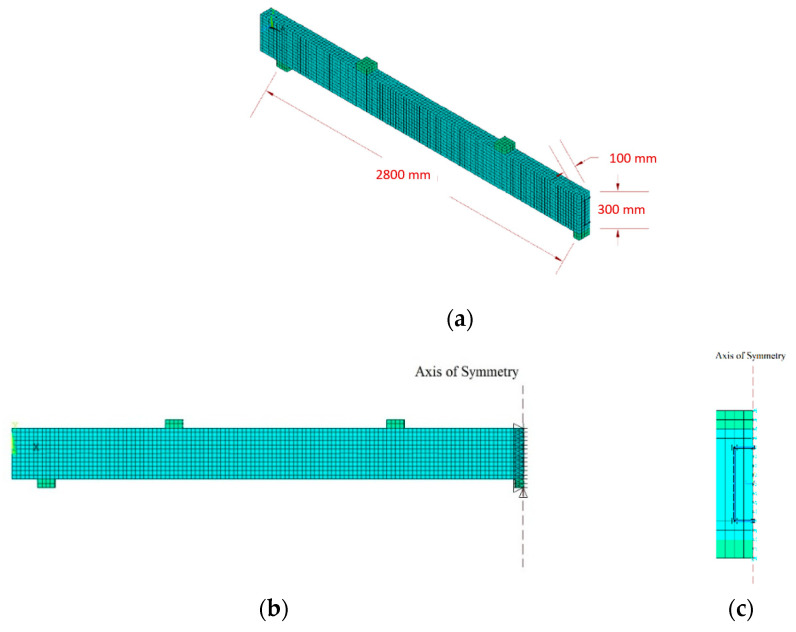
Typical schematic diagrams for (**a**) Isometric view of longitudinal span of beam model, (**b**) Axis of symmetry at middle support with appropriate boundary conditions, and (**c**) Axis of symmetry along the beam’s cross-section with boundary conditions.

The reinforcement configuration and concrete strength varied according to the parameter in question. Only a quarter of the beam was modeled as the beam specimen was symmetrical in the x and y directions. The first axis of symmetry in the x-direction was at the middle support, separating two identical spans at the middle support, so a single span was only modeled. Moreover, half of the beam cross-section was modeled with a width of 100 mm and an axis of symmetry running in the y-direction. Figure 4a presents an isometric view of the typical modeled beam specimen with appropriate boundary conditions assigned to the symmetry axes. Figure 4b,c show the symmetrical axis of the identical modeled beams longitudinally along the beam span and vertically through the cross-section.

### 4.1. Convergence and Meshing

A convergence study was conducted using mesh sizes of 50, 25 and 10 mm. The appropriate mesh size was selected after comparing the deflection values, longitudinal tensile strains, and compressive strains between three identical 50, 25 and 10 mm mesh size models. No significant differences at mid-span deflection, longitudinal tensile strains, and compressive strains were denoted when comparing models established using 25 and 10 mm mesh size. On the contrary, when comparing 50 and 25 mm mesh size models, significant differences at mid-span deflection were observed. Thus, a 25 mm mesh size was selected to reduce the number of elements and the time required for analyzing each beam specimen. 

### 4.2. Solution Procedure

The total applied load was applied in a series of increments. At the end of each load increment, the model’s stiffness matrix was updated, taking into account the nonlinear behavior of structure and materials. Newton Raphson equilibrium iterations were used to update the stiffness matrix. The out-of-balance load vector was evaluated, which is the difference between forces corresponding to internal stresses and the applied external load. If the difference was within the tolerance limit, convergence was accomplished, and the load was iteratively further incremented [30]. Because capturing the post-peak behavior of structure was important to this study and the highly nonlinear behavior of bond-slip behavior of GFRP bars modeled with COMBIN39 elements, a displacement-controlled procedure was chosen to analyze the beams in this study.

## 5. Model Validation and Results

### 5.1. Model Validation 

A finite element model (FEM) was established for verification based on the literature review of Mahmoud [25]. The details of element type and numbers are given in Table 2. Two beam specimens from the existing experimental program were selected to verify the established FEM, Beams GN-1.2-0.48-*d* and GH-1.2-0.63-*d*. In this study, the beam specimens included the two main parameters. Figure 3 shows the beam specimens details and experimental setup for these beam specimens. As shown in Figure 3, the continuous beam comprises two equal spans with hinged support separating the two spans in the middle. Each span is loaded with equal two-point loads with a shear span-to-depth ratio of 3. The continuous beams were reinforced using GFRP bars with equal top and bottom reinforcement. The longitudinal reinforcement ratio for both specimens was 1.2, while the transversal reinforcement ratio was 0.48 and 0.63 for normal and high concrete strength specimens, respectively. The concrete grade used in these two beam specimens, one normal strength of exactly 43 MPa and the other specimen of high strength concrete, was 80 MPa in compressive strengths. Finally, it should be noted that the depth was constant for the two specimens. The material modelling was used as mentioned in the previous section, while the validation model was established. 

### 5.2. Model Validation Results

**Figure 5:**
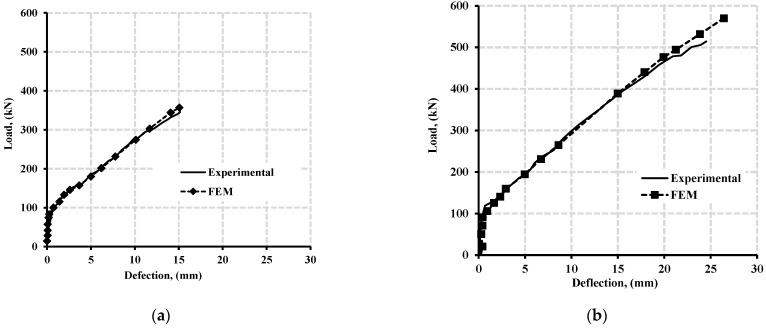
Mid-span deflection of experimental against verified FEM for (**a**) beams specimen GN-1.2-0.48 with normal concrete strength (43 MPa), and (**b**) beams specimen GH-1.2-0.63 with high concrete strength (80 MPa).

**Figure 6:**
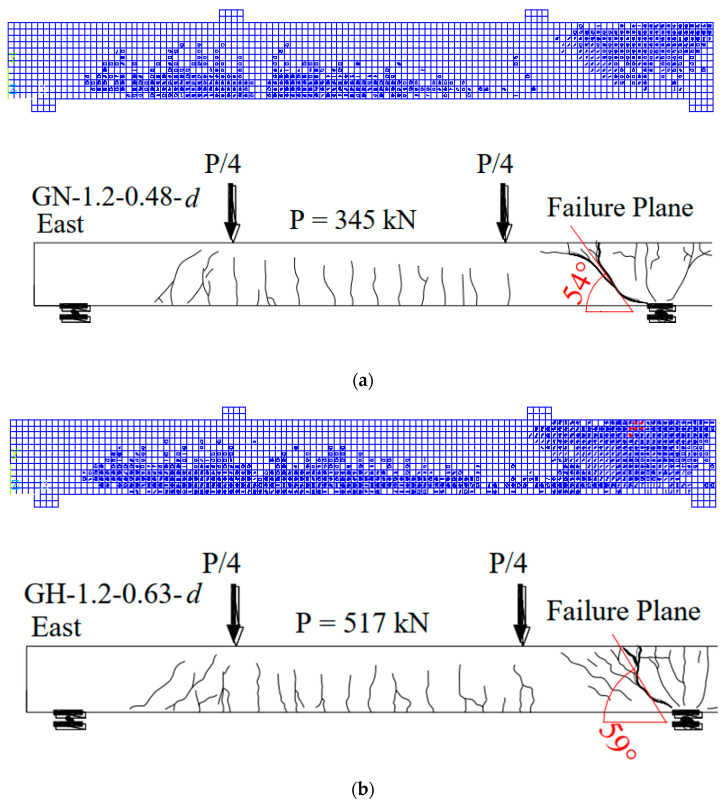
The 3rd crack pattern and mode of failure in comparison to experimental crack pattern from Mahmoud [25] for beam specimens (**a**) GN-1.2-0.48 and (**b**) GH-1.2-0.63.

**Figure 7:**
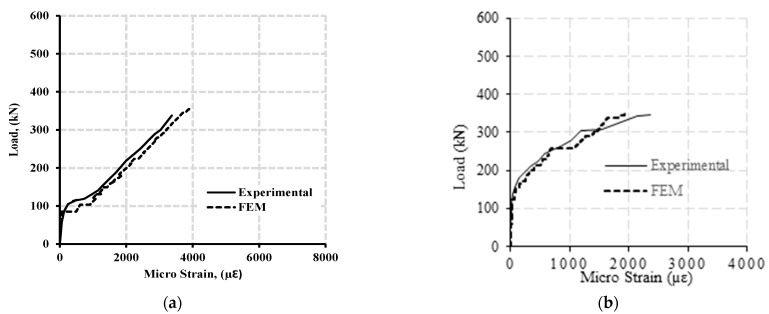
Typical load—strain curve at the hogging moment section of beam specimen GN-1.2-0.48 (with normal concrete strength of 43 MPa) for (**a**) the longitudinal reinforcement and (**b**) stirrup.

**Figure 8:**
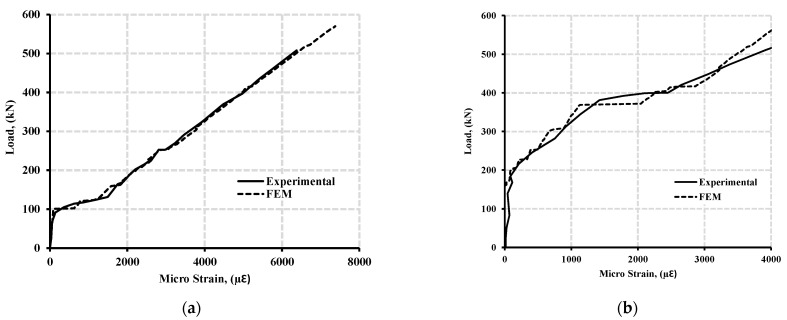
Typical load—strain curve at the hogging moment section of beam specimen GH-1.2-0.63 (with high concrete strength of 80 MPa) for (**a**) the longitudinal reinforcement and (**b**) stirrup.

The experimental and FEM results of the two-beam specimens in mid-span deflection, longitudinal strains at the hogging moment, and the transverse strain of stirrups were compared in Figure 5, Figure 6, Figure 7 and Figure 8. The experimental failure load for beam specimens GN1.2-0.48-*d* and GH-1.2-0.63-*d* provided 345 kN and 517 kN values, as Mahmoud [25] reported. In contrast, the FEM for both specimens provided values of 356.9 kN and 586.8 kN at failure, respectively. Thus, the Exp/FEM ratio resulted in values of 0.97 and 0.88, respectively. Figure 5a,b presents the load-mid span deflection of those two-beam specimens,. The resulting FEM curves of the two specimens were in good agreement with those of the experimental results. At failure, experimentally, both beam specimens, GN-1.20-0.48-*d*, and GH-1.2-0.63-*d* had a mid-span deflection of 15.1 mm and 25.5 mm. While, the FEMs reported 15.2 mm and 22.5 mm, at failure for beam specimens, GN-1.20-0.48-*d*, and GH-1.2-0.63-*d*.

Furthermore, the two-beam specimens were observed to have a similar moment redistribution. The moment distribution observed about 21.5% from the FEM of beam specimen GN-1.2-0.48-*d*, while the experimental results achieved 24% at failure load. For high strength concrete presented in beam specimen GH-1.2-0.63-*d*, the result showed about 20.2% moment distribution compared to that achieved experimentally of 23% at failure load. Regarding the failure mode of beam specimens, GN-1.2-0.48-*d* and GH-1.2-0.63-*d* failed due to diagonal tension cracking at the middle support, according to the reported experimental results by Mahmoud [25]. From the established FEMs, the beam specimen GN-1.2-0.48-*d* failed in diagonal tension after a diagonal crack at interior support intersecting the bent portion of the stirrups, causing brittle failure. On the contrary, the modelled beam specimen GH-1.2-0.63-*d* failed in web crushing at the middle support. The stirrups at the interior shear region underwent large strains until the diagonal crack extended to the compression zone of the concrete at the top fiber of the beam specimen, then failed by crushing the web. This behavior could attribute to the slight difference between the diagonal cracking angles at interior support for both beam specimens, causing diagonal tension that did not pass through the bent portion zone of the stirrups. Thus, this action allowed the straight portion to resist the more significant loading and straining action at interior stirrups. 

Furthermore, the FEMs provided cracks through every SOLID65 element as these elements cracked in three stages by the order during loading having independent orientation. Figure 6a,b shows the third stage of concrete crack that occurred in the SOLID65 results in ANSYS [20] compared with the experimental cracking pattern from Mahmoud [25] for both beams specimens, GN-1.2-0.48-*d* and GH-1.2-0.63-*d*, at failure. As shown in Figure 5, both beam specimens underwent extensive flexural and shear cracking; however, beam GH-1.2-0.63-*d* continued until crushing failure at the elements near the interior shear crack. 

Figure 7a,b shows the load—strain curve of longitudinal and transverse reinforcement at the hogging moment section of beam specimen GN-1.2-0.48-*d* with concrete strength of 43 MPa, while Figure 8a,b shows the load—strain curve of longitudinal and transverse reinforcement at the hogging moment section of beam specimen GH-1.2-0.63-*d* with concrete strength of 80 MPa.

The FEMs for the two-beam specimens, GN-1.2-0.48-*d*, and GH-1.2-0.63-*d*, provided a good agreement with the experimental values regarding failure loads, both reinforcement strains (longitudinal and transverse), mid-span deflection, and moment distribution. These observations confirmed the reliability of the material properties assigned for modelling the continuous beams reinforced with longitudinal and transverse GFRP rebars using normal and high concrete strength. Thus, with the observations mentioned above, the FEM created could accurately predict the behavior of the continuous concrete beams reinforced with longitudinal and transverse GFRP reinforcement and could be reliably used to conduct a parametric study.

## 6. Parametric Studies

Based on the CAN/CSA S806 [16] code, the proposed provision (Equations (7)–(13)) could be used when concrete strength reached 80 MPa. Thus, an upper limit was assigned when calculating the shear resistance of beams. For this reason, six beam specimens with a range from 30 to 80 MPa concrete strength were modelled for this parametric study. Each was a 10 MPa increment higher than the previous. For instance, the concrete strength of the first beam specimen was of concrete strength 30 MPa, and the second one was of 40 MPa, and so on. As per CAN/CSA S806 [16], the longitudinal reinforcement was set by 1.71%, twice the minimum shear reinforcement ratio for normal concrete strength. The percentage was reduced when the concrete reached 1.21% for longitudinal reinforcement, twice the minimum shear reinforcement ratio. The longitudinal bottom and top reinforcement of beam specimens were of three GFRP bars of diameter 19.1 mm. A double branched stirrup configuration was used along the length of the beam specimen of a diameter 12.7 mm bars spaced at 115 mm. 

Moreover, the transverse reinforcement effect was investigated through various models for beam specimens in which the spacing of the stirrups was changed from 115 to two-thirds and halved the later spacing. Thus, reducing the shear reinforcement ratio should allow the beam to undergo more deformations after shear cracking at supports before its failure and, hence, more moment redistribution. It should be mentioned that the top and bottom longitudinal reinforcement were equal in bar diameter 15.9 mm and reinforcement ratio of 0.8%. The stirrups diameter was constant at a 6.3 mm value for the beam specimens. The concrete strength of the beam specimens, in this case, was changed from normal to high strength; 40 MPa presents the normal, and 80 MPa represents the high concrete strength. For high concrete strength beam specimens, the spacing of the initial stirrups was 150 mm instead of 115 mm for those of normal concrete strength beam specimens. The beam specimens of normal concrete strength had a compressive strength of 40 MPa, while high strength specimens had 80 MPa.

Finally, the effect of the longitudinal reinforcement ratio on normal and high concrete strength concrete beams was investigated. Current FRP design recommendations CSA/S806 [16] obligate compressive failure mode in concrete rather than tensile failure mode in reinforcement, and a longitudinal reinforcement ratio ranged from 0.4% to 1.6%, corresponding to c/d ratios of 0.17 to 0.30 in the normal concrete strength at 40 MPa and 0.14 to 0.25 in the high concrete strength at 70 MPa, respectively.

Dimension, reinforcement details, and test setup for typical beams specimen used here in this study are shown in Figure 3. The mechanical bar properties from the area, elastic modulus, tensile strength, and ultimate strain are provided in Table 1. The beam specimens were designed to undergo shear failure before flexural. Table 3 presents the calculated shear strengths, ultimate moment, and corresponding shear force at the ultimate moment according to CSA/S806 [16]. It could be observed that the 24 beams investigated in this parametric study except one had their calculated shear strengths at a lower value than the corresponding shear force at their flexural capacity with the assumption of a 20% moment redistribution, indicating that these beams should undergo shear failure first.

## 7. Results and Discussions

### 7.1. Effect of Concrete Compressive Strength

**Figure 9:**
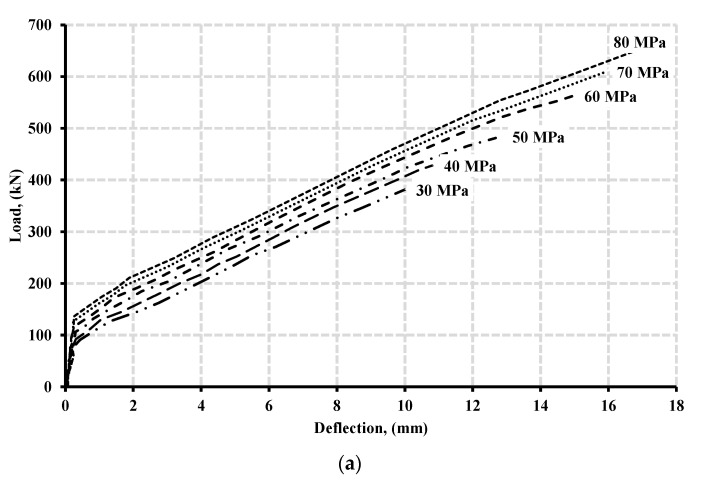

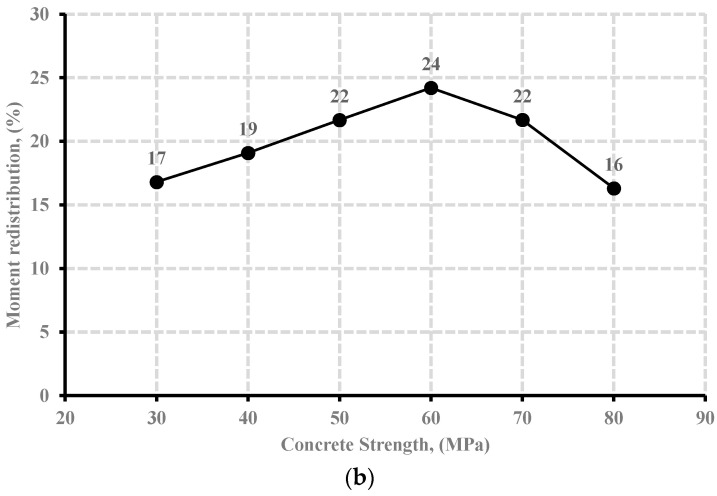
(**a**) Load-deflection curve with varied concrete strength and (**b**) Moment redistribution under variable concrete strength.

Figure 9a illustrates the load-deflection curves at the mid-span of continuous beams reinforced with longitudinal and transverse GFRP bars for concrete strength ranging from 30 to 80 MPa at a 10 MPa increment. Generally, the behavior for beam specimen underwent two phases. The first phase consisted of linearly elastic behavior, where the tensile stresses did not yet exceed the tensile strength capacity of concrete. This phase was characterized by its high stiffness and low deflection relative to other phases. The second phase underwent post-cracking of concrete as the tensile stresses exceed the tensile strength capacity of concrete, creating a crack and reducing its stiffness.

Consequently, increasing the concrete compressive strength enhanced the cracking load capacity of the beam specimens. Furthermore, increasing the concrete compressive strength resulted in higher load capacity of beam specimens with more significant mid-span deflection until failure. These observations agreed with the findings of El-Mogy et al. [8] and Tahenni et al. [31]. Their results revealed a similar enhancement in load capacity and more significant deflection at mid-span at failure when investigating the influence of varying the concrete strength on beams reinforced by GFRP bars.

Figure 9b illustrates the calculated moment redistribution percentages in the hogging moment region based on exterior reactions from the results for each beam specimen where concrete strength varied from 30 to 80 MPa at every 10 MPa increment. At each 10 MPa increment increasing in concrete strength, the moment redistribution percentage enhanced by an average of 2% until the concrete strength reached 60 MPa. On the contrary, after reaching the 60 MPa concrete strength, a reduction in moment redistribution occurred.

**Figure 10:**
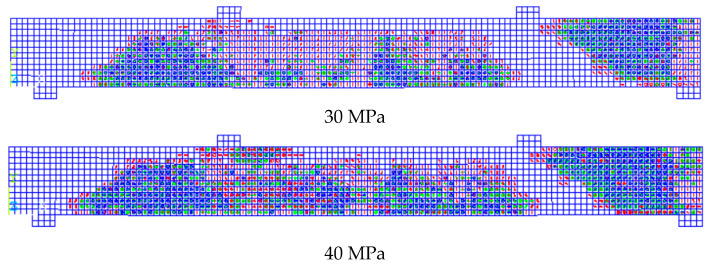

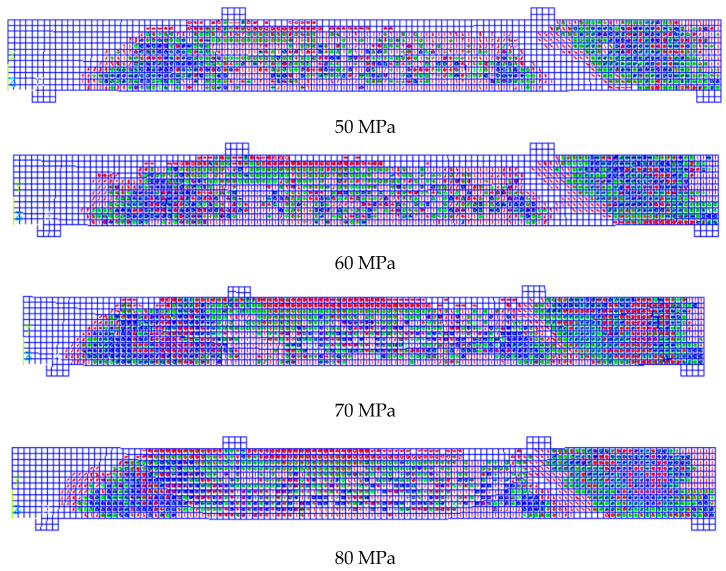
Presents a concrete crack-crush plot for beam specimens with variable concrete strength ranging from 30 to 80 MPa.

The latter behavior could illustrate the reason for those beam specimens of 70 and 80 MPa concrete strength in which no increase in total capacity was observed and, therefore, no moment redistribution occurred, contrary to those beam specimens with lower concrete strength. The results showed that increasing the concrete compressive strength from 50 to 60 MPa resulted in a 14% enhancement in beam capacity. Increasing strength from 60 to 70 and 70 to 80 only resulted in 10% and 7.5% enhancement in the total capacity of the beam specimens, respectively. Figure 10 presents the crack and crush plots for the beam specimens with variable concrete strength at the point of failure.

### 7.2. Effect of Stirrup Spacing

**Figure 11:**
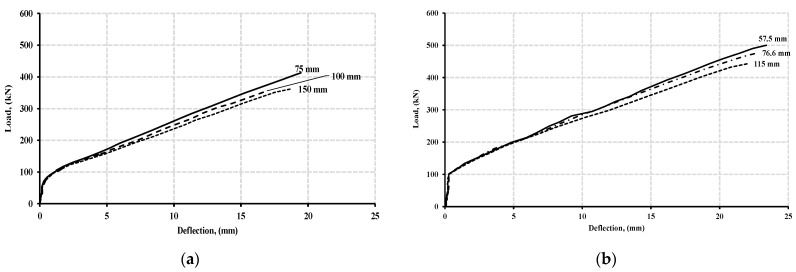
Load-deflection curve for varied stirrups spacing at mid-span for beam specimens with a concrete strength of (**a**) 40 MPa, and (**b**) 80 MPa.

Figure 11a,b demonstrate the load-deflection curves at mid-span for normal and high-strength concrete beam specimens at varied stirrups spacing. These figures showed that the influence of stirrup spacing on load-deflection curves was negligible until the cracking phase. For high-strength concrete beam specimens, the cracking load was approximately 105 kN, while for those of normal-strength beam specimens, the cracking load reached 70 kN, regardless of the stirrup spacing. At the post-cracking phase, beam specimens with smaller stirrup spacing had more stiffness and therefore underwent lesser deflection. The inversely proportional relationship between stirrup spacing and post-cracking flexural stiffness was more noticeable in normal concrete strength beam specimens than high-strength concrete counterparts’ specimens. At the failure stage, halving stirrup spacing from 150 mm to 75 mm in normal concrete strength beam specimens provided a higher load-deflection ratio, from 19.2 at 150 mm stirrups spacing to 21.2 at 75 mm stirrups spacing. Thus, the increase in mid-span stiffness reached 10%. In high-strength concrete specimens, halving the stirrup spacing from 115 mm to 57.5 mm provided a larger load-deflection ratio from 20.1 to 21.4, providing about 6% in mid-span stiffness. The increase in stiffness resulting from FRP stirrups was consistent with Hussein et al. [32] observation, who modelled FRP reinforced beams using ANSYS.

**Figure 12:**
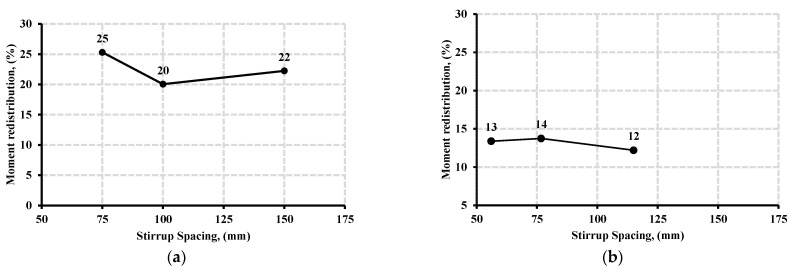
The relationship between the moment redistribution and stirrup spacing for beams specimens with concrete strength of (**a**) 40 MPa, and (**b**) 80 MPa.

Figure 12a,b show the respective moment redistribution of beam specimens at two-thirds and halves of the initial stirrups spacing for normal and high concrete strength. From Figure 12a,b, it was observed that the moment redistribution percentage was not directly proportional to the variation in stirrup spacing. For normal strength concrete specimens, the average moment redistribution across the beam specimens was 23.3%. The reduction in stirrups spacing from 150 to 75 mm caused an overall increase in moment redistribution by 13.7%. For high strength, concrete specimens were less responsive to the variation of stirrup spacing.

**Figure 13:**
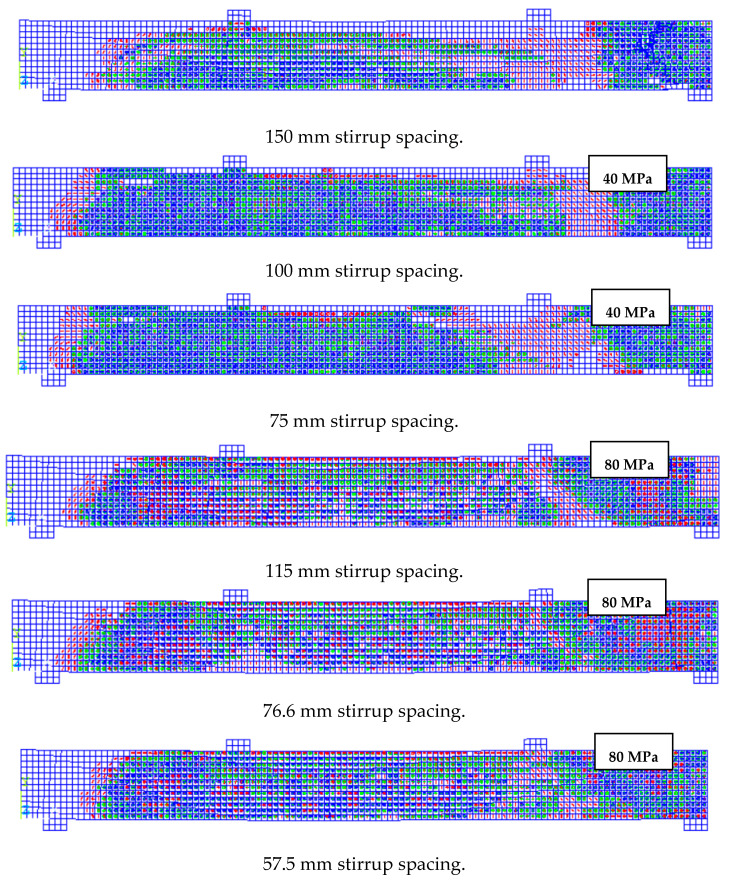
Presents concrete crack-crush plot for beams with variable stirrup spacing for normal and high strength concrete beam specimens.

**Figure 14:**
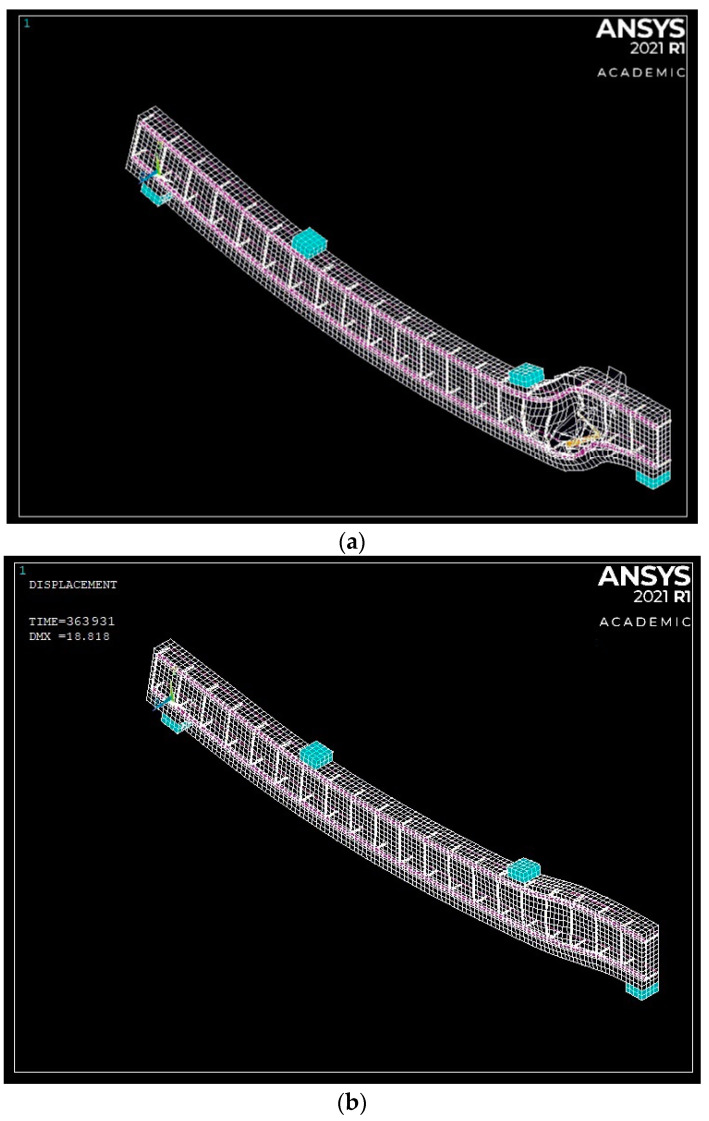
Typical 3D deflection shapes: (**a**) typical shear failure and (**b**) typical flexure failure.

The average moment redistribution was 13% across the beam specimens. The reduction in stirrups spacing from 115 mm to 57.5 mm resulted in an increase of 8.3% of moment redistribution for the beam specimens. The result showed that normal strength concrete beam specimens exhibited higher moment redistribution than those of high strength concrete counterparts specimens; however, both groups exhibited a redistribution of the moment when reducing the spacing of the stirrups. These findings were in good agreement with those of El-Mogy et al. [8]. Their experimental results showed a similar redistribution of the moment at normal concrete strength specimens when the spacing of the stirrups was varied while investigating various parameters that influence the continuous reinforced concrete beams with FRP bars, longitudinally and transversely. Figure 13 and Figure 14 presents the crack and crush plots for beam specimens with variable stirrup spacing at the point of failure.

### 7.3. Effect of Longitudinal Reinforcement Ratio

**Figure 15:**
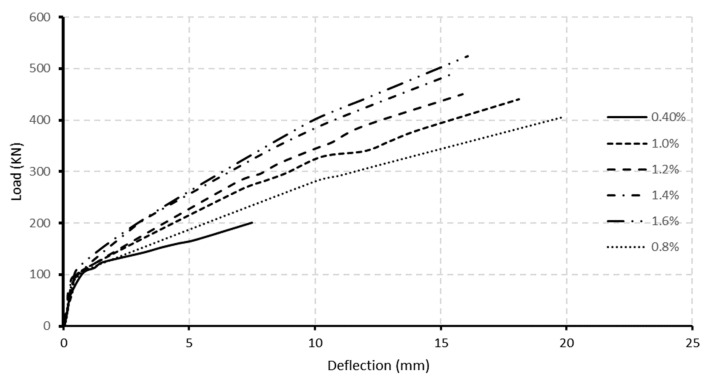
Load–deflection curve for variable longitudinal reinforcement ratio for normal concrete strength beam specimens (40 MPa).

**Figure 16:**
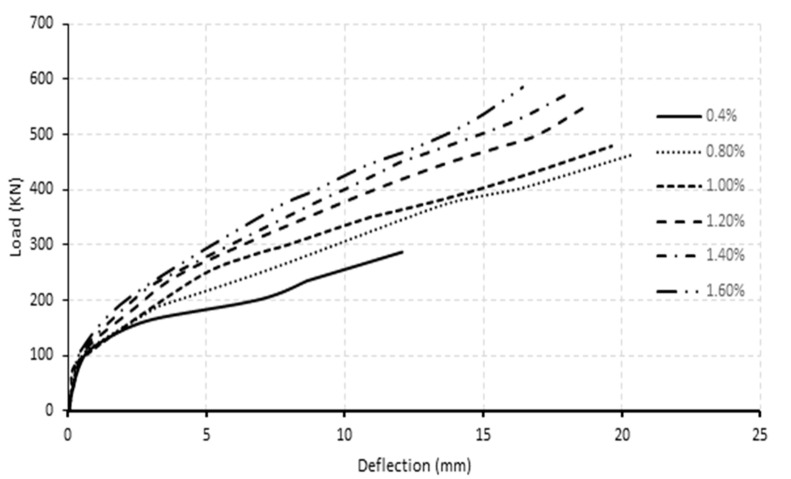
Load–deflection curve for variable longitudinal reinforcement ratio for normal concrete strength beam specimens (80 MPa).

Figure 15 and Figure 16 depict the load-deflection relationship for models with longitudinal reinforcement ratios ranging from 0.4% to 1.6%. As could be observed, the model with a 0.4% reinforcement ratio at the middle support had the lowest post-cracking flexural stiffness and ultimate load capacity. The post-cracking flexural stiffness and ultimate load improved significantly as the reinforcing ratio increased at mid-span and middle support. This behavior represents the impact of raising the longitudinal reinforcement ratio’s axial stiffness. On the contrary, raising the reinforcement ratio improved the final load capacity. This behavior might result from the beam’s shear capacity due to an increase in ultimate failure load. In normal and high concrete strength models, increasing the reinforcement ratio from 0.4% to 0.8% increased the ultimate load capacity by approximately 98% and 59%, respectively, while increasing the longitudinal reinforcement ratio from 0.8 to 1.6 percent increased the load capacity by approximately 30 and 28 percent for normal and high concrete strength beams.

**Figure 17:**
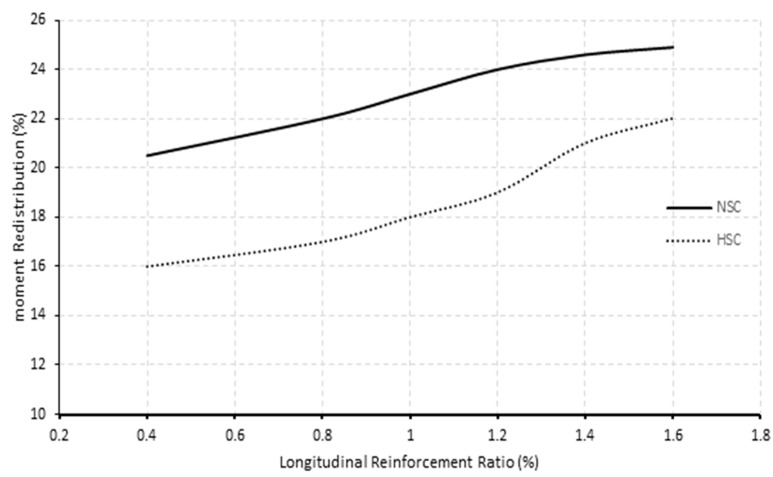
The moment redistribution and longitudinal ratio relationship for normal (40 MPa) and high (80 MPa) concrete strength beam specimens.

The link between the longitudinal reinforcement ratio and the moment redistribution at the middle support is shown in Figure 17. A clear trend of increasing moment redistribution for normal and high concrete strength beams increased the longitudinal reinforcement ratios. Increasing longitudinal reinforcement ratio from 0.4% to 1.2% resulted in an increase in moment redistribution of 17% and 19%. Further increments from 1.2% to 1.6% did not significantly gain moment redistribution for normal concrete strength beams. Finally, across the reinforcement ratios studied, normal concrete strength beams consistently redistributed more moments than those high concrete strength beams counterparts. 

## 8. Shear Capacity Provisions

### 8.1. CAN/CSA S806 Provisions

The CSA S806 [16] specifies that the nominal shear resistance, *V_r_*, of FRP-reinforced concrete members can be computed as the sum of concrete and stirrup contribution resistance as:

(7) $$V_{r}=V_{c}+V_{sF}\leq 0.22f^{\prime }b_{w}d_{v}$$

The effective depth of cross-section, *d_v_*, was calculated using the depth of the longitudinal reinforcement (from the extreme top fiber of the beam till the centroid of the bar) multiplied by 0.9. The concrete contribution *V_c_* for sections with an effective depth not exceeding 300 mm and with no axial load acting on them could be calculated using the following Equations (8)–(10):

(8) $$V_{c}=0.05\lambda \phi_{c}k_{m}k_{r}{\left(f^{\prime }\right)}^{1/3}b_{w}d_{v}$$

(9) $$k_{m}={\left(V_{f}d/M_{f}\right)}^{1/2}$$

(10) $$k_{r}=1+{\left(E_{Fl}\rho_{Fl}\right)}^{1/3}$$

where *λ* is the concrete density factor and equal to 1.0 for normal density concrete, $\phi_{c}$ presents the material resistance factor, and $k_{m}$ and $k_{r}$ are factors accounting for the ratio of the moment provided by shear force to the total moment of the section and the effect of both longitudinal FRP reinforcement ratios $\rho_{Fl}$ and elastic modulus *E_fl_* on the shear strength of the section in consideration. The vertical stirrup contribution in a concrete member, $V_{sF}$, could be calculated using the following Equations (11)–(13):

(11) $$V_{sF}=\frac{A_{v}f_{v}d_{v}}{s}\cot\theta$$

(12) $$\theta =30+7000\varepsilon_{1}$$

(13) $$\varepsilon_{1}=\frac{\frac{M_{f}}{d_{v}}+V_{f}+0.5N_{f}}{2E_{F}A_{F}}$$

*A_v_* presents stirrup reinforcement, and $\varepsilon_{1}$ is the average longitudinal strain at mid-height of the section of interest. The maximum stress $f_{v}$ in Equation (11) could be determined by the smaller value of $\;0.005E_{F}$, $\;0.4f_{fu}.\;$ Alternatively, the maximum stress could be equal to 1200 MPa, while θ presents the angle of the diagonal tension crack.

### 8.2. Oller’s Model Provision

For comparison purposes, a literature review of other models was used. Oller et al. [33] had proposed a shear model that accounts for the main effective parameters contributing to shear through collecting a database on the simply supported beams reinforced with longitudinal and transverse FRP reinforcement. The results revealed a good agreement with the results of the database set collected from the literature review (more than 121 beams). According to Oller et al. [33], the total shear resisted by any section reinforced longitudinally and transversely by FRP reinforcement should be the sum of three components, as shown in Equation (14):

(14) $$V=f_{ct}\cdot b\cdot d\cdot \left(v_{c}+v_{w}+v_{t}\right)$$

*_Vc_* presents the concrete contribution, *v_w_* presents the shear transferred across the crack, and *v_t_* shows the vertical FRP stirrups contribution. Finally,$\;f_{ct}$ provides the rupture strength of concrete. For beams reinforced with longitudinally FRP bars, the concrete contribution calculated using Equations (15) and (16):

(15) $$v_{c}=\zeta \cdot \left(1.072-0.01\cdot \alpha \right)\cdot \left(\left(0.98+0.22\cdot v_{t}\right)\cdot \xi +0.05\right)$$

(16) $$\zeta =1.2-0.2\cdot a=1.2-0.2\cdot \frac{a}{d}\cdot d\geq 0.65$$

The concrete contribution is the function of beam size and depends mainly on the shear span of the beam in meters, as presented by the factor ζ. At the same time, ξ represents the relative neutral axis depth c/d for the beam’s cross-section. On the other hand, the contribution of FRP stirrups resisting the shear capacity of the beam could be evaluated as follows:

(17) $$v_{t}=\frac{\rho_{t}\cdot 0.85\cdot E_{t}\cdot \varepsilon_{t}}{f_{ct}}$$

(18) $$\varepsilon_{t}=0.225\cdot \varepsilon_{tu}$$

where $\rho_{t}$ is the transverse reinforcement ratio; *E_t_* and *ε_t_* are the elastic modulus of the stirrup reinforcement and the mean strain in the stirrups, respectively. The mean strain of the stirrups could be obtained as a fraction of the ultimate rupture strain of FRP stirrups, $\varepsilon_{tu}$. By assuming a mean crack angle of 41.4° degrees, the tensile strength transferred across the crack is presented by *v_w_*, which could be obtained from Equation (18) as follows:

(19) $$v_{w}=\frac{0.386}{\varepsilon_{t}}\cdot \frac{f_{ct}}{E_{c}}\left(1+\frac{8\cdot G_{f}\cdot E_{c}}{f_{ct}^{2}\cdot d}\right)$$

The *G_f_* simulated the fracture energy of concrete and evaluated it as a function of concrete rupture strength and ultimate tensile strain.

## 9. Comparison between Analytical and Finite Element Models

Table 4 presented the shear strength for beams with variable concrete strengths at the hogging section and predicted the shear capacity of the beam as calculated per CSA/S806 [16]. It could be seen that CSA/S806 [16] underestimated the capacity of the beam specimens. The prediction was closest to the lower concrete strength and began to deviate with the increase in the concrete strength. The average ratio of shear strength resulting from the FEM model, and that predicted by CSA/S806 [16] was 1.16 ± 0.20, with a variance coefficient of 17.22%. These results are close to the findings of Razaqpur et al. [34], who also assessed CSA/S806 [16] shear provisions for beams reinforced with FRP. In contrast, the model developed by Oller et al. [33] overestimated the capacity of the beams, especially the beams with low concrete strength resulting in a V_FEM_/V_Pred_ ratio of 0.76 ± 0.12 and a variance coefficient of 15%.

Table 5 presents the shear strength for beams with variable stirrup spacing at the hogging section, and the predicted shear capacity of the beam was calculated according to CSA/S806 [16] for normal and high strength concrete specimens, respectively. The mean ratio of shear strength from the FEM model predicted by CSA/S806 [16] for normal-strength concrete beams was 1.60 ± 0.14, with a coefficient of variation equal to 9%. The high-strength concrete beams yielded a similar result with a mean ratio of 1.50 ± 0.15 and a coefficient of variation valued at 10%. On the other hand, the analytical model suggested by Oller et al. [33] yielded better results with mean ratios of 1.20 ± 0.25 and 1.27 ± 0.28, and the coefficient of variation valued at 20% and 22% for normal strength and high strength concrete beam specimens at the spacing of the varied stirrups as assigned.

Table 6 presents the shear strength for beams with variable longitudinal reinforcement ratio calculated from CSA/S806 [16] and Oller et al. [33] for normal and high strength concrete specimens, respectively. The mean ratio of shear strength from the FEM model predicted by CSA/S806 [16] for normal concrete strength beams is 1.56 ± 0.11, with a coefficient of variation equal to 7%. The high-strength concrete beams yielded a similar result with a mean ratio of 1.52 ± 0.08 and a coefficient of variation valued at 5%. On the other hand, the analytical model suggested by Oller et al. [33] yielded better results with mean ratios of 1.13 ± 0.24 and 1.08 ± 0.18, and the coefficient of variation was valued at 21% and 16% for normal and high concrete strength beam specimens.

## 10. Moment Redistribution Prediction through Empirical Methods

A multivariate linear regression analysis was conducted on the 24 beam specimens in the parametric study using several key parameters that could influence moment redistribution. Parameters with a low statistical significance were discarded to maximize the coefficient of determination. As a result, Equation (19) was formulated:

(20) $$MR\%=-2.81f^{\prime }+4.9\rho_{Fl}+7.927\;\rho_{Ft}-0.001081145\;E_{ft}+0.008987166\;E_{c\;}-74.91\;$$

MR% is the predicted moment redistribution percentage at failure, where $\rho_{Fl}$ and $\rho_{Ft}$ are longitudinal and transversal reinforcement ratios, respectively. $f^{\prime }$, $E_{ft},$ and $E_{c}$ are the concrete compressive strength, the elastic modulus of GFRP transverse reinforcement, and concrete in MPa. The equation has a coefficient of determination or an R-squared value of 0.718 and a standard error of 2.03. It is also worth noting that concrete compressive strength and concrete elastic modulus had the most significant influence on moment redistribution of beams with *p* values of 0.0003 and 0.0005 that were significantly less than the *p* values of the rest of the parameters.

## 11. Conclusions

The numerical investigation was initiated by creating an FEM to simulate the behavior of continuously supported concrete beams reinforced with longitudinal and transversal GFRP bars reinforcement. The FEM was verified against the experimental results shown by Mahmoud [25]. A parametric study was conducted utilizing the verified model to investigate the influence of various concrete compressive strengths and GFRP stirrups spacing on moment redistribution of continuous beams. The conclusions based on the results and discussions limited to this parametric numerical program were presented as follow:

1. The numerical simulation using the finite element model by ANSYS software adequately predicted the behavior of continuously supported GFRP reinforced beams. The results extracted from the FEMs were in good agreement regarding ultimate failure loads, deflection behavior, and moment redistribution against the experimental data provided by Mahmoud [25].
2. Despite the absence of plastic behavior in GFRP reinforcement, moment redistribution still occurs on GFRP reinforcement beams at the post cracking stage and the degree of redistribution affected by GFRP reinforcement.
3. At failure, the two FEMs established for simulating beam specimens, GN-1.2-0.48-*d*, and GH-1.2-0.63-*d* were in good agreement with their experimental results. The moment distribution observed about 21.5% from the FEM of beam specimen GN-1.2-0.48-*d*, while the experimental results achieved 24% at failure load. For high strength concrete presented in beam specimen GH-1.2-0.63-*d*, the result showed about 20.2% moment distribution as compared to that achieved experimentally of 23% at failure load.
4. The increase in concrete strength resulted in an enhancement in moment distribution. The behavior was limited only to beams that had a concrete strength not exceeding 60 MPa. High-strength concrete specimens with concrete strengths more than 75 MPa generally distributed less moment than their normal strength concrete counterparts.
5. The reduction in stirrups spacing from 150 to 75 mm caused an overall increase in moment redistribution by 13.7% at normal concrete strength. The reduction in stirrups spacing from 115 mm to 57.5 mm, resulted in an increase of 8.3% of moment redistribution for the beam specimens at high concrete strength.
6. Stirrup spacing impacted the moment redistribution in continuous beams. However, its ability to influence moment redistribution seemed to diminish with high concrete strengths. In addition, the relationship between spacing and moment redistribution was not directly proportional. Reducing the shear reinforcement ratio by reducing the stirrups’ spacing while maintaining constant stirrup diameter did not always enhance moment redistribution.
7. Limited to this study, the CSA S806 [16] had a mean FEM to predicted shear strength ratio of 1.42 with a coefficient of variation valued 12.1%, while Oller et al. [33] had a much better mean ratio of 1.08 with a slightly higher variance coefficient of 19%.
8. This study has shown that the GFRP reinforcement can be effectively modelled in ANSYS software. The finite element analysis results were found to be in close agreement with the experimental results. The finite element analysis is always considered a more affordable and faster analysis approach as compared to the labor-intensive investigations (experimental research). Therefore, the outcome of this study is very useful, and the proposed analysis can be further used for the economical and safe design of GFRP reinforced concrete structures.

## Figures and Tables

**Table 1:** Properties of the GFRP bars.

Bar Type	Designated Diameter	Nominal Cross-Sectional Area (mm^2^)	CSA/S806(2012) Annex A (mm^2^)	Tensile Strength, *f_fu_*, (MPa)	Modulus of Elasticity, *E_f_*, (GPa)	Ultimate Strain (%)
GFRP	15.9	198	282.7	1442	67	2.1
	19.1	285	396.6	1383	53	2.6
	6.3 *	32 *	44.0 *	1383 *	53 *	2.6 *
	9.5 *	72 *	84.7 *	1215 *	53 *	2.7 *
	12.7 *	127 *	145.7 *	1328 *	53 *	2.5 *

* For straight portions of the stirrup.

**Table 2:** Element type and numbers.

Element Type	Element Count	Node Count	Behavior Modelled
Solid 45	68	210	Loading Plates
Solid 65	5760	7865	Concrete
Link 180	480	484	Longitudinal Reinforcement
Link 180	294	315	Stirrup reinforcement
Combin39	1600	3200	Bond-slip behavior and bar shear strength
Total	8205	12,074	

**Table 3:** The failure mode, bending moment, and shear capacity at ultimate as per CSA/S806 [16].

Parameter			Ultimate Moment CSA/S806 (kN·m)	Shear Force at Ultimate Moment (kN)	Shear Strength CSA/S806 (kN)	Failure Mode
Variable Concrete Strength, (MPa)	Concrete Strength	30	79.3	120.2	130.7	flexural
		40	93.6	141.9	135.1	Shear
		50	105.4	159.7	138.5	
		60	115.2	174.6	139.4	
		70	123.4	187.0	141.9	
		80	130.3	197.5	144.2	
Variable Stirrup Spacing, (mm)	40 MPa	150	70.9	107.5	75.4	Shear
		100	70.9	107.5	78.3	
		75	70.9	107.5	78.7	
	80 MPa	115	94.1	142.5	91.4	
		76.6	94.1	142.5	92.4	
		57.5	94.1	142.5	93.4	
Variable Longitudinal Reinforcement Ratio, (%)	40 MPa	0.4	53.6	81.3	45.5	Shear
		0.8	70.9	107.5	75.0	
		1.0	77.2	117.0	83.2	
		1.2	82.6	125.2	90.0	
		1.4	87.3	132.3	95.7	
		1.6	91.5	138.7	100.8	
	80 MPa	0.4	70.0	106.1	54.4	Shear
		0.8	94.1	142.5	92.5	
		1.0	103.0	156.1	102.9	
		1.2	110.8	167.9	111.4	
		1.4	117.7	178.3	118.7	
		1.6	123.9	187.7	125.1	

**Table 4:** FEM results and predicted shear strength for beam specimens with variable compressive strength.

Concrete Strength (MPa)	Model Shear Strength (kN)	Predicted Shear Strength Using CSA/S806, (kN)	V_FEM_/V_Pred_	Predicted Shear Strength Using Oller et al. [33], (kN)	V_FEM_/V_Pred_
30	118.6	130.7	0.91	194	0.61
40	132.5	135.1	0.98	201	0.66
50	148.9	138.5	1.08	208	0.72
60	171.9	139.4	1.23	213	0.81
70	187.2	141.9	1.32	219	0.86
80	203.9	144.2	1.41	224	0.91
Mean			1.16		0.76
SD			0.20		0.12
COV (%)			17%		15%

**Table 5:** FEM results and predicted shear strength for beam specimens of concrete strength 40 and 80 MPa with variable stirrup spacing.

Series By Concrete Strength, (MPa)	Stirrup Spacing (mm)	Model Shear Strength (kN)	Predicted Shear Strength Using CSA/S806 (kN)	V_FEM_/V_Pred_	Predicted Shear Strength Using Oller et al. [33], (kN)	V_FEM_/V_Pred_
40	150	125.8	75.4	1.67	79.8	1.58
	100	109.9	78.3	1.4	92.9	1.18
	75	111.5	78.7	1.42	105.9	1.05
	Mean			1.50		1.27
	SD			0.15		0.28
	COV (%)			10%		22%
80	115	157	91.4	1.72	108.06	1.45
	76.6	149.2	92.4	1.62	125.13	1.19
	57.7	135.9	93.4	1.45	142.1	0.96
	Mean average:			1.60		1.20
	SD			0.14		0.25
	COF-V (%):			9%		20%

**Table 6:** FEM results and predicted shear strength for beam specimens of concrete strength 40 and 80 MPa with variable longitudinal reinforcement ratio.

Series By Concrete Strength, (MPa)	Longitudinal Reinforcement Ratio (%)	Model Shear Strength (kN)	Predicted Shear Strength Using CSA/S806, (kN)	V_FEM_/V_Pred_	Predicted Shear Strength Using Oller et al. [33], (kN)	V_FEM_/V_Pred_
40	0.4	62.0	45.5	1.36	96.1	0.65
	0.8	124.9	75.0	1.67	107.6	1.16
	1.0	135.1	83.2	1.62	112.0	1.21
	1.2	137.8	90.0	1.53	115.8	1.19
	1.4	149.3	95.7	1.56	119.3	1.25
	1.6	160.3	100.8	1.59	122.4	1.31
	Mean			1.56		1.13
	SD			0.11		0.24
	COV (%)			7%		21%
80	0.4	89.5	54.4	1.65	120.0	0.75
	0.8	143.5	92.5	1.55	133.2	1.08
	1.0	148.3	102.9	1.44	138.3	1.07
	1.2	170.1	111.4	1.53	142.8	1.19
	1.4	176.8	118.7	1.49	146.8	1.20
	1.6	180.2	125.1	1.44	150.5	1.20
	Mean average:			1.52		1.08
	SD			0.08		0.18
	COF-V (%):			5%		16%

## Data Availability

The data presented in this study are available on request from the corresponding author.
